# Supplementary material for: A Likelihood Approach for Real-Time Calibration of Stochastic Compartmental Epidemic Models
Source: PLoS Comput Biol. 2017 Jan 17;13(1):e1005257. doi: 10.1371/journal.pcbi.1005257 (PMC5240920; doi:10.1371/journal.pcbi.1005257)
Supplement: S1 File — (TAR.GZ) [file pcbi.1005257.s014.tar.gz › HSPH_Online-SI-Revision/PF08/n100-extreme/n100-extreme_PF2_table8.pdf]

1	newly 3 weeks spec	655.	1452.32	{1399., 1501.}	121.728	0.345674	newly 3 weeks cumul	2650.	5752.93	{5694., 5837.}	117.092	0.336618	newly 5 weeks spec	298.	859.98	{806., 901.}	188.584	0.459914	newly 5 weeks cumul	3391.	7732.42	{7619., 7842.}	128.028	0.35796	newly 46 weeks cumul	4195.	10482.5	{10315., 10677.}	149.882	0.397694
2	newly 3 weeks spec	761.	1459.03	{1416., 1501.}	91.7254	0.282546	newly 3 weeks cumul	3077.	5539.37	{5475., 5602.}	80.025	0.255314	newly 5 weeks spec	441.	933.84	{893., 972.}	111.755	0.325603	newly 5 weeks cumul	4068.	7642.14	{7574., 7703.}	87.8599	0.273823	newly 42 weeks cumul	5247.	11202.1	{11059., 11344.}	113.495	0.329364
3	newly 3 weeks spec	277.	1022.91	{983., 1063.}	269.282	0.567161	newly 3 weeks cumul	1321.	4142.81	{4086., 4199.}	213.612	0.496367	newly 5 weeks spec	122.	574.55	{545., 603.}	370.943	0.67262	newly 5 weeks cumul	1607.	5486.65	{5430., 5548.}	241.422	0.533277	newly 47 weeks cumul	1853.	7228.2	{7140., 7306.}	290.081	0.591139
4	newly 3 weeks spec	75.	1394.61	{1354., 1437.}	1759.48	1.26928	newly 3 weeks cumul	495.	5710.58	{5645., 5766.}	1053.65	1.06206	newly 5 weeks spec	26.	785.28	{746., 820.}	2920.31	1.47974	newly 5 weeks cumul	564.	7543.75	{7497., 7601.}	1237.54	1.1263	newly 51 weeks cumul	589.	9860.31	{9802., 9913.}	1574.08	1.22377
5	newly 3 weeks spec	383.	1412.72	{1371., 1455.}	268.856	0.566734	newly 3 weeks cumul	1884.	5673.31	{5612., 5729.}	201.131	0.478738	newly 5 weeks spec	164.	818.15	{782., 857.}	398.872	0.697715	newly 5 weeks cumul	2324.	7556.64	{7489., 7613.}	225.157	0.512082	newly 48 weeks cumul	2679.	10076.6	{9976., 10172.}	276.133	0.575328
6	newly 3 weeks spec	620.	1640.92	{1591., 1693.}	164.665	0.42255	newly 3 weeks cumul	2591.	6468.01	{6386., 6555.}	149.634	0.397283	newly 5 weeks spec	303.	979.53	{939., 1026.}	223.277	0.509296	newly 5 weeks cumul	3314.	8723.65	{8631., 8810.}	163.236	0.420329	newly 46 weeks cumul	4005.	11903.5	{11730., 12068.}	197.215	0.473044
7	newly 3 weeks spec	425.	2487.09	{2409., 2561.}	485.198	0.767199	newly 3 weeks cumul	2093.	9089.89	{9012., 9166.}	334.3	0.637779	newly 5 weeks spec	238.	1673.89	{1612., 1735.}	603.315	0.846986	newly 5 weeks cumul	2658.	12806.6	{12712., 12907.}	381.812	0.682869	newly 44 weeks cumul	3121.	19697.	{19498., 19876.}	531.111	0.800094
8	newly 3 weeks spec	611.	1647.92	{1597., 1698.}	169.709	0.430793	newly 3 weeks cumul	2634.	6453.21	{6374., 6521.}	144.997	0.389143	newly 5 weeks spec	315.	984.75	{945., 1030.}	212.619	0.494766	newly 5 weeks cumul	3371.	8717.01	{8636., 8799.}	158.588	0.412594	newly 46 weeks cumul	4133.	11977.	{11794., 12136.}	189.79	0.462057
9	newly 3 weeks spec	509.	2061.18	{2000., 2115.}	304.947	0.607297	newly 3 weeks cumul	2442.	7890.58	{7808., 7970.}	223.12	0.50935	newly 5 weeks spec	256.	1276.32	{1230., 1322.}	398.563	0.69754	newly 5 weeks cumul	3053.	10795.4	{10698., 10879.}	253.6	0.548503	newly 46 weeks cumul	3616.	15214.5	{15070., 15392.}	320.755	0.624015
10	newly 3 weeks spec	32.	1258.81	{1215., 1303.}	3833.78	1.59466	newly 3 weeks cumul	165.	5145.81	{5095., 5197.}	3018.67	1.49396	newly 5 weeks spec	7.	703.84	{674., 738.}	9954.86	2.00213	newly 5 weeks cumul	184.	6790.36	{6740., 6836.}	3590.41	1.56707	newly 50 weeks cumul	192.	8869.	{8808., 8929.}	4519.27	1.66457
11	newly 3 weeks spec	582.	1210.87	{1159., 1258.}	108.053	0.317937	newly 3 weeks cumul	2565.	4780.31	{4693., 4867.}	86.3669	0.270328	newly 5 weeks spec	318.	726.42	{688., 766.}	128.434	0.358393	newly 5 weeks cumul	3307.	6446.72	{6332., 6570.}	94.9416	0.289861	newly 44 weeks cumul	4044.	8855.72	{8672., 9040.}	118.984	0.340349
12	newly 3 weeks spec	488.	2524.78	{2466., 2589.}	417.373	0.713721	newly 3 weeks cumul	2113.	9333.1	{9236., 9413.}	341.699	0.645115	newly 5 weeks spec	223.	1658.62	{1599., 1710.}	643.776	0.871282	newly 5 weeks cumul	2630.	13043.5	{12944., 13134.}	395.952	0.695432	newly 45 weeks cumul	3083.	19562.4	{19363., 19735.}	534.525	0.802439
13	newly 3 weeks spec	1177.	2067.99	{2002., 2122.}	75.7001	0.24466	newly 3 weeks cumul	4644.	7567.2	{7485., 7647.}	62.9457	0.21203	newly 5 weeks spec	679.	1396.81	{1335., 1444.}	105.716	0.313096	newly 5 weeks cumul	6187.	10669.4	{10583., 10750.}	72.4488	0.23665	newly 43 weeks cumul	8302.	16539.7	{16358., 16731.}	99.2252	0.299329
14	newly 3 weeks spec	9.	3248.14	{3176., 3318.}	35990.4	2.55732	newly 3 weeks cumul	64.	12076.5	{11965., 12174.}	18769.6	2.27575	newly 5 weeks spec	5.	2072.86	{2011., 2129.}	41357.2	2.6175	newly 5 weeks cumul	74.	16765.	{16649., 16886.}	22555.4	2.35517	newly 48 weeks cumul	86.	24200.1	{23992., 24361.}	28039.7	2.44931
15	newly 3 weeks spec	73.	1314.4	{1272., 1352.}	1700.55	1.25528	newly 3 weeks cumul	382.	5315.11	{5254., 5372.}	1291.39	1.14343	newly 5 weeks spec	19.	755.12	{717., 789.}	3874.32	1.59896	newly 5 weeks cumul	449.	7072.91	{7011., 7124.}	1475.26	1.19734	newly 48 weeks cumul	476.	9398.01	{9339., 9453.}	1874.37	1.29542
16	newly 3 weeks spec	111.	1467.45	{1423., 1509.}	1222.03	1.12114	newly 3 weeks cumul	653.	5935.76	{5873., 5996.}	808.998	0.958548	newly 5 weeks spec	31.	833.63	{799., 863.}	2589.13	1.42938	newly 5 weeks cumul	748.	7876.07	{7818., 7933.}	952.951	1.0224	newly 48 weeks cumul	812.	10403.1	{10331., 10481.}	1181.17	1.1076
17	newly 3 weeks spec	848.	1659.95	{1611., 1713.}	95.7488	0.29159	newly 3 weeks cumul	3379.	6282.57	{6216., 6341.}	85.9299	0.269333	newly 5 weeks spec	459.	1067.16	{1020., 1111.}	132.497	0.366172	newly 5 weeks cumul	4480.	8676.08	{8586., 8756.}	93.6625	0.287035	newly 43 weeks cumul	5685.	12706.6	{12574., 12839.}	123.511	0.349285
18	newly 3 weeks spec	371.	2688.3	{2611., 2748.}	624.609	0.860023	newly 3 weeks cumul	1789.	9475.39	{9377., 9553.}	429.647	0.723976	newly 5 weeks spec	168.	1911.55	{1853., 1966.}	1037.83	1.05594	newly 5 weeks cumul	2207.	13663.2	{13582., 13754.}	519.085	0.791744	newly 44 weeks cumul	2601.	22455.9	{22239., 22645.}	763.354	0.936177
19	newly 3 weeks spec	522.	2548.06	{2483., 2615.}	388.134	0.688456	newly 3 weeks cumul	2300.	9439.48	{9353., 9513.}	310.412	0.613209	newly 5 weeks spec	225.	1673.97	{1616., 1727.}	643.987	0.871418	newly 5 weeks cumul	2858.	13187.6	{13088., 13284.}	361.427	0.664095	newly 46 weeks cumul	3344.	19719.3	{19548., 19911.}	489.692	0.770615
20	newly 3 weeks spec	31.	2016.93	{1966., 2066.}	6406.23	1.81326	newly 3 weeks cumul	168.	8152.79	{8071., 8217.}	4752.85	1.68599	newly 5 weeks spec	4.	1147.9	{1106., 1196.}	28597.5	2.45765	newly 5 weeks cumul	185.	10823.1	{10744., 10900.}	5750.32	1.76717	newly 51 weeks cumul	187.	14268.7	{14198., 14335.}	7530.32	1.88254
21	newly 3 weeks spec	203.	1289.58	{1242., 1336.}	535.261	0.8028	newly 3 weeks cumul	1027.	5226.29	{5166., 5279.}	408.889	0.706606	newly 5 weeks spec	76.	740.41	{701., 773.}	874.224	0.98837	newly 5 weeks cumul	1233.	6940.73	{6887., 6999.}	462.914	0.750433	newly 48 weeks cumul	1341.	9188.05	{9121., 9253.}	585.164	0.835788
22	newly 3 weeks spec	138.	1158.71	{1124., 1196.}	739.645	0.923963	newly 3 weeks cumul	788.	4741.43	{4688., 4796.}	501.704	0.779366	newly 5 weeks spec	76.	648.56	{612., 683.}	753.368	0.930774	newly 5 weeks cumul	953.	6255.35	{6200., 6308.}	556.385	0.817149	newly 49 weeks cumul	1046.	8154.72	{8102., 8214.}	679.61	0.891871
23	newly 3 weeks spec	646.	2382.86	{2320., 2437.}	268.864	0.566779	newly 3 weeks cumul	2717.	8444.72	{8374., 8523.}	210.81	0.492484	newly 5 weeks spec	343.	1690.85	{1639., 1745.}	392.959	0.692676	newly 5 weeks cumul	3563.	12149.7	{12040., 12253.}	240.996	0.53274	newly 42 weeks cumul	4442.	20129.2	{19886., 20325.}	353.157	0.656231
24	newly 3 weeks spec	888.	2508.47	{2447., 2577.}	182.485	0.45092	newly 3 weeks cumul	3507.	8840.43	{8752., 8929.}	152.08	0.401525	newly 5 weeks spec	441.	1788.82	{1737., 1848.}	305.628	0.607994	newly 5 weeks cumul	4589.	12756.6	{12647., 12867.}	177.982	0.444007	newly 42 weeks cumul	5895.	21275.7	{21050., 21483.}	260.91	0.557381
25	newly 3 weeks spec	135.	3044.2	{2955., 3113.}	2154.96	1.35306	newly 3 weeks cumul	801.	10887.5	{10783., 10989.}	1259.24	1.13328	newly 5 weeks spec	46.	2111.24	{2048., 2165.}	4489.65	1.66165	newly 5 weeks cumul	936.	15546.3	{15436., 15656.}	1560.93	1.22034	newly 45 weeks cumul	1018.	24558.2	{24310., 24809.}	2312.4	1.38244
26	newly 3 weeks spec	1048.	1504.46	{1464., 1549.}	43.5553	0.156886	newly 3 weeks cumul	4173.	5744.02	{5661., 5820.}	37.6473	0.138736	newly 5 weeks spec	644.	949.71	{910., 987.}	47.4705	0.168487	newly 5 weeks cumul	5651.	7889.86	{7809., 7968.}	39.6188	0.144926	newly 43 weeks cumul	7533.	11366.3	{11225., 11531.}	50.8861	0.178624
27	newly 3 weeks spec	806.	1108.63	{1065., 1160.}	37.5471	0.138226	newly 3 weeks cumul	3332.	4382.03	{4316., 4448.}	31.5135	0.118925	newly 5 weeks spec	451.	661.92	{623., 693.}	46.7672	0.166167	newly 5 weeks cumul	4401.	5900.06	{5817., 5987.}	34.0618	0.12725	newly 44 weeks cumul	5811.	8062.73	{7891., 8223.}	38.7494	0.14213
28	newly 3 weeks spec	568.	947.44	{906., 978.}	66.8028	0.221996	newly 3 weeks cumul	2482.	3873.4	{3811., 3926.}	56.0596	0.193261	newly 5 weeks spec	266.	532.49	{501., 559.}	100.184	0.301036	newly 5 weeks cumul	3132.	5114.48	{5037., 5184.}	63.2976	0.212955	newly 48 weeks cumul	3853.	6690.58	{6579., 6786.}	73.646	0.239637
29	newly 3 weeks spec	769.	2399.98	{2337., 2453.}	212.091	0.494198	newly 3 weeks cumul	3241.	8548.73	{8478., 8620.}	163.768	0.42121	newly 5 weeks spec	412.	1683.28	{1628., 1737.}	308.563	0.611119	newly 5 weeks cumul	4213.	12249.5	{12154., 12361.}	190.754	0.463517	newly 42 weeks cumul	5339.	20039.1	{19774., 20314.}	275.334	0.574398
30	newly 3 weeks spec	278.	1411.71	{1367., 1450.}	407.809	0.705578	newly 3 weeks cumul	1314.	5757.67	{5701., 5815.}	338.179	0.641637	newly 5 weeks spec	87.	795.39	{763., 831.}	814.241	0.960787	newly 5 weeks cumul	1559.	7610.04	{7550., 7668.}	388.136	0.688533	newly 51 weeks cumul	1722.	9973.13	{9900., 10048.}	479.16	0.762792
31	newly 3 weeks spec	58.	1674.27	{1625., 1722.}	2786.67	1.46029	newly 3 weeks cumul	360.	6799.22	{6721., 6867.}	1788.67	1.27614	newly 5 weeks spec	22.	948.8	{904., 989.}	4212.73	1.63451	newly 5 weeks cumul	413.	9006.06	{8948., 9061.}	2080.64	1.33858	newly 52 weeks cumul	442.	11793.1	{11732., 11858.}	2568.11	1.4262
32	newly 3 weeks spec	101.	1126.92	{1081., 1164.}	1015.76	1.04741	newly 3 weeks cumul	549.	4619.29	{4570., 4666.}	741.401	0.924989	newly 5 weeks spec	30.	631.57	{601., 664.}	2005.23	1.323	newly 5 weeks cumul	633.	6095.27	{6047., 6138.}	862.918	0.983582	newly 49 weeks cumul	682.	7973.7	{7909., 8028.}	1069.16	1.06787
33	newly 3 weeks spec	918.	1186.9	{1135., 1236.}	29.2919	0.111351	newly 3 weeks cumul	3832.	4803.08	{4670., 4942.}	25.3413	0.0979811	newly 5 weeks spec	479.	678.74	{638., 721.}	41.6994	0.150941	newly 5 weeks cumul	4959.	6380.39	{6201., 6602.}	28.6628	0.109338	newly 47 weeks cumul	6261.	8467.91	{8215., 8706.}	35.2485	0.131011
34	newly 3 weeks spec	843.	1668.72	{1613., 1716.}	97.9502	0.296404	newly 3 weeks cumul	3518.	6235.35	{6124., 6343.}	77.2413	0.248518	newly 5 weeks spec	471.	1095.01	{1045., 1142.}	132.486	0.366158	newly 5 weeks cumul	4638.	8691.56	{8556., 8863.}	87.3989	0.27273	newly 42 weeks cumul	6028.	13073.6	{12808., 13370.}	116.881	0.336162
35	newly 3 weeks spec	693.	1788.5	{1730., 1837.}	158.081	0.411621	newly 3 weeks cumul	3121.	7092.42	{7015., 7177.}	127.248	0.356486	newly 5 weeks spec	338.	1060.69	{1019., 1100.}	213.814	0.496447	newly 5 weeks cumul	3977.	9533.74	{9456., 9627.}	139.722	0.379696	newly 47 weeks cumul	4760.	12861.3	{12701., 13011.}	170.195	0.431659
36	newly 3 weeks spec	635.	1051.47	{1001., 1098.}	65.5858	0.218717	newly 3 weeks cumul	2904.	4243.23	{4136., 4385.}	46.1167	0.164594	newly 5 weeks spec	364.	604.17	{559., 647.}	65.9808	0.219387	newly 5 weeks cumul	3816.	5649.39	{5499., 5833.}	48.0448	0.170272	newly 45 weeks cumul	4844.	7497.6	{7273., 7798.}	54.7812	0.189553
37	newly 3 weeks spec	121.	1286.85	{1244., 1329.}	963.512	1.02662	newly 3 weeks cumul	634.	5263.73	{5203., 5319.}	730.241	0.919188	newly 5 weeks spec	49.	724.36	{684., 754.}	1378.29	1.16944	newly 5 weeks cumul	759.	6954.48	{6893., 7014.}	816.269	0.962014	newly 50 weeks cumul	824.	9113.31	{9055., 9167.}	1005.98	1.04374
38	newly 3 weeks spec	12.	1794.52	{1743., 1849.}	14854.3	2.17466	newly 3 weeks cumul	113.	7287.45	{7212., 7351.}	6349.07	1.80949	newly 5 weeks spec	3.	1027.27	{985., 1068.}	34142.3	2.53433	newly 5 weeks cumul	121.	9676.26	{9607., 9736.}	7896.91	1.90292	newly 50 weeks cumul	126.	12748.1	{12684., 12811.}	10017.5	2.00507
39	newly 3 weeks spec	302.	1690.55	{1637., 1742.}	459.785	0.747879	newly 3 weeks cumul	1471.	6494.89	{6420., 6566.}	341.529	0.644941	newly 5 weeks spec	115.	1056.79	{1011., 1108.}	818.948	0.963042	newly 5 weeks cumul	1796.	8889.5	{8788., 8985.}	394.961	0.694556	newly 44 weeks cumul	2002.	12666.3	{12519., 12846.}	532.683	0.801166
40	newly 3 weeks spec	529.	984.91	{867., 1093.}	86.1834	0.268033	newly 3 weeks cumul	2325.	3957.35	{3471., 4335.}	70.2086	0.229109	newly 5 weeks spec	275.	568.2	{484., 647.}	106.618	0.312344	newly 5 weeks cumul	2963.	5275.83	{4613., 5805.}	78.057	0.248628	newly 45 weeks cumul	3697.	7060.22	{6151., 7808.}	90.9716	0.278927
41	newly 3 weeks spec	253.	820.2	{772., 857.}	224.19	0.510319	newly 3 weeks cumul	1228.	3410.24	{3337., 3466.}	177.707	0.443442	newly 5 weeks spec	114.	445.16	{413., 474.}	290.491	0.590824	newly 5 weeks cumul	1511.	4461.07	{4364., 4536.}	195.24	0.470021	newly 46 weeks cumul	1685.	5682.09	{5519., 5788.}	237.216	0.527691
42	newly 3 weeks spec	23.	1270.06	{1227., 1304.}	5422.	1.74196	newly 3 weeks cumul	166.	5197.73	{5142., 5258.}	3031.16	1.49569	newly 5 weeks spec	8.	717.52	{684., 748.}	8869.	1.95247	newly 5 weeks cumul	186.	6867.35	{6820., 6913.}	3592.12	1.56727	newly 50 weeks cumul	187.	8987.87	{8923., 9057.}	4706.35	1.68181
43	newly 3 weeks spec	681.	3132.	{3059., 3204.}	359.912	0.662603	newly 3 weeks cumul	3118.	10671.8	{10567., 10768.}	242.264	0.534351	newly 5 weeks spec	377.	2337.03	{2271., 2403.}	519.902	0.792204	newly 5 weeks cumul	4043.	15728.8	{15617., 15821.}	289.037	0.589985	newly 43 weeks cumul	5018.	27667.3	{27361., 27912.}	451.361	0.741424
44	newly 3 weeks spec	174.	1114.17	{1079., 1148.}	540.328	0.806273	newly 3 weeks cumul	824.	4561.89	{4503., 4613.}	453.627	0.7432	newly 5 weeks spec	61.	628.3	{596., 663.}	930.	1.01248	newly 5 weeks cumul	988.	6022.91	{5970., 6065.}	509.606	0.78504	newly 49 weeks cumul	1084.	7894.26	{7831., 7953.}	628.253	0.862274
45	newly 3 weeks spec	45.	1452.98	{1405., 1495.}	3128.84	1.50893	newly 3 weeks cumul	244.	5868.18	{5812., 5929.}	2304.99	1.3811	newly 5 weeks spec	9.	825.49	{789., 860.}	9072.11	1.96221	newly 5 weeks cumul	276.	7784.72	{7726., 7835.}	2720.55	1.45033	newly 48 weeks cumul	280.	10296.3	{10228., 10369.}	3577.23	1.56551
46	newly 3 weeks spec	223.	1595.58	{1539., 1649.}	615.507	0.854465	newly 3 weeks cumul	1040.	6406.89	{6340., 6477.}	516.047	0.789598	newly 5 weeks spec	61.	923.81	{879., 971.}	1414.44	1.17997	newly 5 weeks cumul	1223.	8542.54	{8479., 8613.}	598.491	0.84415	newly 48 weeks cumul	1322.	11398.6	{11309., 11510.}	762.226	0.935612
47	newly 3 weeks spec	132.	1255.1	{1214., 1294.}	850.833	0.977966	newly 3 weeks cumul	614.	5145.55	{5081., 5210.}	738.037	0.923241	newly 5 weeks spec	43.	709.67	{681., 738.}	1550.4	1.21738	newly 5 weeks cumul	740.	6796.95	{6738., 6847.}	818.507	0.963073	newly 50 weeks cumul	804.	8886.12	{8821., 8967.}	1005.24	1.04345
48	newly 3 weeks spec	186.	1530.7	{1488., 1576.}	722.957	0.915254	newly 3 weeks cumul	969.	6276.06	{6202., 6336.}	547.684	0.81135	newly 5 weeks spec	49.	866.87	{830., 900.}	1669.12	1.24754	newly 5 weeks cumul	1115.	8297.	{8231., 8353.}	644.126	0.871639	newly 50 weeks cumul	1182.	10847.3	{10777., 10919.}	817.707	0.962699
49	newly 3 weeks spec	8.	1710.44	{1655., 1752.}	21280.5	2.32991	newly 3 weeks cumul	68.	6972.03	{6912., 7033.}	10153.	2.01084	newly 5 weeks spec	3.	970.12	{931., 1004.}	32237.3	2.50951	newly 5 weeks cumul	72.	9228.66	{9164., 9284.}	12717.6	2.1078	newly 52 weeks cumul	76.	12094.6	{12023., 12168.}	15814.	2.20177
50	newly 3 weeks spec	733.	1808.89	{1759., 1863.}	146.779	0.392211	newly 3 weeks cumul	2944.	6767.07	{6692., 6844.}	129.86	0.361449	newly 5 weeks spec	408.	1182.7	{1132., 1226.}	189.877	0.461988	newly 5 weeks cumul	3889.	9423.61	{9337., 9502.}	142.314	0.384369	newly 43 weeks cumul	4985.	14102.4	{13960., 14241.}	182.896	0.451612
